# Power Laws in Empirical Eigenvalue Spectra

**DOI:** 10.3390/e28040418

**Published:** 2026-04-09

**Authors:** Benyuan Liu, Yung-Ying Chen, M. Shane Li, Vanessa Thomasin Morgan, Eslam Abdelaleem, Audrey Sederberg

**Affiliations:** 1 School of Physics, Georgia Institute of Technology, Atlanta, GA 30332, USA; bliu398@gatech.edu (B.L.); yyc@gatech.edu (Y.-Y.C.); mli853@gatech.edu (M.S.L.); eslam.abdelaleem@gatech.edu (E.A.); 2 School of Psychology, Georgia Institute of Technology, Atlanta, GA 30332, USA; 3 Graduate Program in Neuroscience, University of Minnesota, Minneapolis, MN 55455, USA; morg0448@umn.edu

**Keywords:** phenomenological renormalization group, power-law scaling, spontaneous neural data

## Abstract

The critical brain hypothesis proposes that neural systems operate near a phase transition to optimize information processing. A key method for investigating this hypothesis is the phenomenological renormalization group (pRG), which looks for scale-invariant features across levels of coarse-graining. One such feature is the power-law scaling of eigenvalues of covariance matrices of coarse-grained variables. However, the estimation of this scaling exponent, μ, often relies on linear regression over arbitrarily selected ranges of the plot of eigenvalues versus rank. This heuristic “eyeballing” introduces uncontrolled bias and complicates the interpretation of observed scaling relationships. In order to obtain a more robust estimation of μ, we do not fit the standard eigenvalue-vs-rank relationship, but rather the density of eigenvalues, for which standard protocols exist for fitting power laws to empirical data distributions. We demonstrate this approach using a synthetic model that replicates the scaling signatures of neural data while providing control over the system’s exponents as well as neural data obtained from publicly available Neuropixels recordings. We also establish standards for the minimal data required to quantify power-law behavior in a pRG eigenvalue analysis. Our approach contributes a tool for understanding the fundamental limitations imposed by spatial and temporal constraints of experimental datasets, which is required to rigorously assess the neural criticality hypothesis.

## 1. Introduction

The critical brain hypothesis is a leading theoretical framework to interpret emergent phenomena in neural systems. From the first observations of avalanche scaling in cultured tissue [1] to the recent development of renormalization group-based methods in large-scale neural recordings [2], criticality signatures have been quantified in many different types of neural data (electrophysiology [3,4,5], calcium imaging [2,6,7], fMRI [8,9], MEG [10]) and across species (mice [7,11,12,13], rats [4,14], turtles [15,16], macaques [12,17,18], humans [8,10,19,20]). While a power law alone does not prove a system is critical—judging this requires also analyzing relationships between scaling exponents, universal shape collapse, and so on [21,22]—without a clear estimation of scaling exponents, it is impossible to proceed further in the analysis. Robustly quantifying power laws in experimental datasets of modest size is a major challenge in interpreting scaling phenomena, something that is well-known in the analysis of avalanche scaling [23,24,25]. With new approaches to detecting signatures of criticality in neural activity, this challenge is intensified.

Based on the renormalization group approach developed for physical systems, the phenomenological renormalization group (pRG) uses correlations in neural data as a proxy for distance in order to coarse-grain neural activity across scales [2]. By iteratively combining the most correlated pairs of neurons, the pRG analysis groups neurons in clusters of size $K=2^{k}$, k=0,1,2,…. pRG analysis reveals scaling of cluster variance, covariance, autocorrelation, and activity distributions with cluster size *K* [2,3,12,19]. In particular, the eigenvalues (λ) of the cluster covariance matrix scale with their normalized rank, ${\left(r/K\right)}^{-\mu }$. Different brain areas appear to yield different exponents μ [3]. If robustly estimated, such exponents could provide a compact summary statistic of network organization and potentially link dynamical state to function.

However, estimating μ from finite data is challenging. In practice, exponent estimation often relies on linear regression in log-log coordinates. If points are not appropriately subsampled in log-space or weighted, this approach can yield spuriously high $R^{2}$ values. Even when weighted appropriately, finite-size effects limit the range over which scaling holds, and selecting the cutoff from eigenvalue versus rank plots can be practitioner-dependent. As a result, uncertainty in exponent estimates and goodness-of-fit is rarely quantified systematically. Without such calibration, it is difficult to rigorously compare models or assess the statistical power required to detect scaling. In particular, clear guidelines are lacking for how many neurons and how much recording time are required to reliably estimate a scaling exponent.

In this work, we draw on the insight that rank-scaling may be viewed instead as a scaling in the distribution [3,26,27], for which existing protocols for power-law analysis may be applied [28], yielding both a measure of goodness-of-fit and parameter uncertainty estimation. Our paper is structured as follows. We first illustrate the pRG process for obtaining the covariance matrices whose eigenvalues have approximate power-law distribution. After demonstrating the fitting procedure on a toy model with known cutoff, we examine the reliability of exponent fitting on a simulated neural dataset, systematically varying the population size and the duration of the recording to determine minimum sizes and durations for which exponent estimation is stable. We examine scaling in eleven publicly available recordings from two datasets [29,30], finding that four recordings were statistically compatible with a power-law model, six were narrowly rejected but exhibited near-scaling behavior, and one clearly deviated from power-law structure. Our results have implications for how scaling exponents are measured and interpreted across many neural recordings.

## 2. Methods

Symbols used throughout the paper are defined in Table 1.

### 2.1. Phenomenological Renormalization Group Analysis

**Figure 1:**
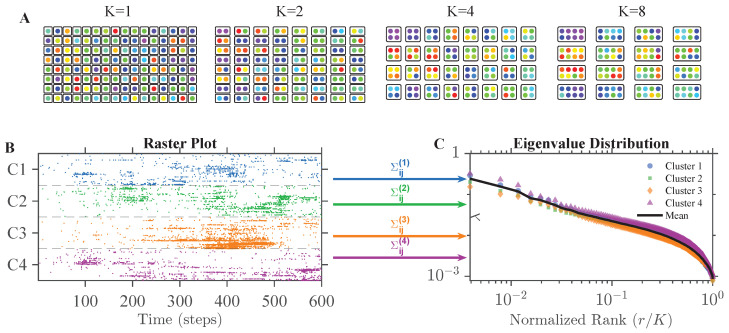
pRG applied on simulated neural data and single-scale (K=256) eigenvalue distribution analysis. (**A**) A schematic of pRG process: neurons are represented by dots. Neurons with more similar colors are more correlated with each other. During coarse-graining, high-correlation pairs are merged into clusters. As we coarse-grain the data, cluster statistics are analyzed at different scales (K=1,2,4,8, etc.). (**B**) An example of synthetic neural data with 1024 neurons and cluster size = 256, C1–C4 represent the four clusters at K=256. (**C**) Eigenvalue distribution in rank/K space of different clusters and mean eigenvalue distribution.

Phenomenological renormalization group (pRG) [2,31] is a correlation-based coarse-graining framework inspired by real-space RG in spin systems [32,33] used to probe multiscale scaling structures in population activity. In pRG, pairwise correlations are used as a proxy for distance, as in neural systems, anatomical proximity does not necessarily reflect functional interactions. Generally, pRG [2] proceeds as follows: The most correlated pair of neurons is selected and merged into a new unit by summing activity and normalizing such that the average non-zero activity level is equal to 1. This process is repeated, moving on to the next most correlated pairs until no further merging is available. By repeating this process, individual neurons (cluster of size 1) merge to form clusters of sizes 2,4,8,…, as illustrated in Figure 1A, where neurons that are highly correlated with each other are represented by dots with similar colors.

These clusters allow us to observe the system at different scales. If scaling phenomena exist, we should observe consistent statistical behaviors across all cluster sizes, including the scaling with *K* of overall cluster activity variance, cluster autocorrelation time, and cluster free energy. Each of these is simple to estimate, as each of these statistics produces a single scalar value at each cluster size *K*. On the other hand, the final scaling relationship is obtained by taking the original activity within each cluster, as shown for clusters of size K=256 in Figure 1B, and computing the eigenvalues of the covariance matrix as in Figure 1C. The averaged eigenvalue λ(r) at each rank *r* is plotted in black in Figure 1C, and these scale approximately as λ(r)∼${\left(r/K\right)}^{-\mu }$.

Due to finite-size effects, the eigenvalue–rank curve typically exhibits an apparent power-law “head” followed by a rapidly decaying tail. Determining the boundary between these regions by visual inspection introduces subjective bias and can substantially affect the estimated exponent. To avoid this ambiguity, we do not fit the eigenvalue–rank relationship directly. Instead, we fit the distribution of eigenvalues, for which established statistical procedures exist for power-law inference.

### 2.2. Estimation of Eigenvalue Scaling Exponent μ

We estimate the scaling exponent using the maximum likelihood estimator (MLE) framework of Clauset et al. [28]. Specifically, we fit the probability density of eigenvalues above a lower cutoff $\lambda_{\min}$, assuming a power-law tail of the form $p\left(\lambda \right)\propto \lambda^{-\alpha }$ for $\lambda \geq \lambda_{\min}$. For each candidate $\lambda_{\min}$, the continuous exponent $\hat{\alpha }$ is derived from the eigenvalues $\lambda_{i}\geq \lambda_{\min}$ via the closed-form Hill estimator [34]:

(1) $$\hat{\alpha }=1+n{\left[\sum_{i=1}^{n}\ln\frac{\lambda_{i}}{\lambda_{\min}}\right]}^{-1}.$$

The optimal lower bound $\lambda_{\min}$ is identified by minimizing the Kolmogorov–Smirnov (KS) distance, denoted as $\hat{D}$, between the empirical eigenvalue distribution and the theoretical power-law cumulative distribution function (CDF) with exponent $\hat{\alpha }$ and cutoff $\lambda_{\min}$.

Crucially, while the MLE procedure will always yield an estimated exponent, it does not guarantee that the empirical data actually follows a power-law distribution. To assess the plausibility of the power-law hypothesis, we perform a bootstrap goodness-of-fit test. We generate a large ensemble of synthetic surrogate datasets drawn from a true power-law distribution configured with our empirically fitted parameters (${\hat{\lambda }}_{\min}$ and $\hat{\alpha }$). Each surrogate dataset is subjected to the exact same fitting procedure as the real data to compute a surrogate KS statistic, $D_{i}$. The *p*-value is defined as the fraction of surrogate datasets for which $D_{i}$ exceeds the empirical distance $\hat{D}$. In this framework, the *p*-value quantifies whether a power law can be rejected: a low *p*-value indicates that the empirical data’s deviation from the model is larger than what would be expected by random chance. Following conservative standard practice, if p<0.1, the power-law hypothesis is statistically rejected.

Finally, to align with the standard phenomenological renormalization group (pRG) literature, we map the probability density scaling exponent α back to the rank-scaling exponent μ (defined by $\lambda \propto r^{-\mu }$). Integrating the PDF to obtain the rank relationship yields the exact transformation μ=1/(α−1). Using the Delta method, the corresponding standard error propagates to a concise analytic form: $\sigma_{\mu }=\mu /\sqrt{n}$, where *n* is the number of eigenvalues in the fitted tail.

A comprehensive mathematical derivation of this estimation, goodness-of-fit quantification, and error propagation procedure is provided in Appendix A.

### 2.3. Real and Synthetic Neural Population Activity

#### 2.3.1. Synthetic Neural Data

We simulated population activity using the dynamical latent variable model described in [25,35]. Neurons are binary units $S_{i}\left(t\right)\in \left\{0,1\right\}$ whose activity is coupled to $N_{F}$ shared latent fields $h_{m}\left(t\right)$ through random weights $J_{im}$∼$N(0,N_{F}^{-1})$. The system is governed by the Hamiltonian

(2) $$H\left(t\right)=-\eta \sum_{i=1}^{N}\sum_{m=1}^{N_{F}}J_{im}S_{i}\left(t\right)h_{m}\left(t\right)+\epsilon \sum_{i=1}^{N}S_{i}\left(t\right),$$

where η controls coupling strength and ϵ sets the mean firing rate. Latent fields evolve independently with autocorrelation time τ. Neuron states are updated via Gibbs sampling according to the energy difference induced by flipping $S_{i}$. Unless otherwise stated, simulations used $N_{F}=10$ latent fields, η=3, ϵ=8, τ=2s, and time step Δt=0.02s. These parameters are chosen to place the system in a regime that exhibits stable, nontrivial correlations and avoids both uncorrelated and saturated dynamics, under which robust power-law scaling is observed. Total duration *T* and population size *N* were varied as described in Section 3.

#### 2.3.2. Experimental Recordings

We used published datasets obtained from Neuropixels recordings in the mouse brain. The datasets are summarized in Table 2. All datasets consist of “spontaneous” activity during which no specific visual (or other) stimuli were provided.

In four datasets [29], eight Neuropixels 1.0 probes were simultaneously inserted to record neural activity across multiple brain regions. Mice were awake and allowed to run freely on a wheel, without engaging in any specific behavioral task.

In seven datasets [30], multiple Neuropixels probes were used to record approximately 30,000 neurons across 42 brain regions while mice performed a visual discrimination task. We only selected spontaneous intervals, defined as periods without task engagement or stimulus presentation, for the following analysis.

In both experiment cases, we restricted our analysis to brain regions with at least N=128 simultaneously recorded neurons. Following Morales et al. [3], we defined a characteristic timescale for each session as the geometric mean of the inter-spike intervals across all neurons, and used this value to set the width of the time bins for discretizing spiking activity.

## 3. Results

We address the problem of fitting an apparent power law in eigenvalue distributions generated by the pRG analysis. We first illustrate the method to estimate the scaling of eigenvalues by rank, demonstrating how the fit range and the uncertainty of the parameters are determined using a toy model with known ground-truth (Section 3.1). We then demonstrate the fitting method on a surrogate neural dataset, in which we can vary the overall population size and the overall recording duration, to establish minimal requirements for attempting a pRG eigenvalue analysis (Section 3.2). Finally, we apply our methods to multiple real datasets and discuss the results (Section 3.3).

### 3.1. Fitting Eigenvalue vs. Rank in a Toy Model: The Perils of Cutoff Estimation

To illustrate the challenge of fitting the approximate power-law relationship of eigenvalue λ versus rank *r*, λ(r)∼$r^{-\mu }$, we first analyzed the problem in a toy model [28] with a modification specific to pRG eigenvalue analysis. In this model, the density of eigenvalues ρ(λ) is modeled by:

(3) $$\rho \left(\lambda \right)=\left\{\begin{array}{cc} C{\left(\lambda /\lambda_{\min}\right)}^{-\alpha } & \mathrm{for}\;\lambda \geq \lambda_{\min},\\ Ce^{-\alpha (\lambda /\lambda_{\min}-1)} & \mathrm{for}\;\lambda <\lambda_{\min}, \end{array}\right.$$

with the scaling parameter α>1 and cutoff $\lambda_{\min}$. The normalization constant *C* is determined by $\int_{0}^{\infty }\rho \left(\lambda \right)d\lambda =1$. This distribution is continuous at $\lambda_{\min}$ by construction. In this example, we use α=2.6 and $\lambda_{\min}=0.99$, which gives C=1.

**Figure 2:**
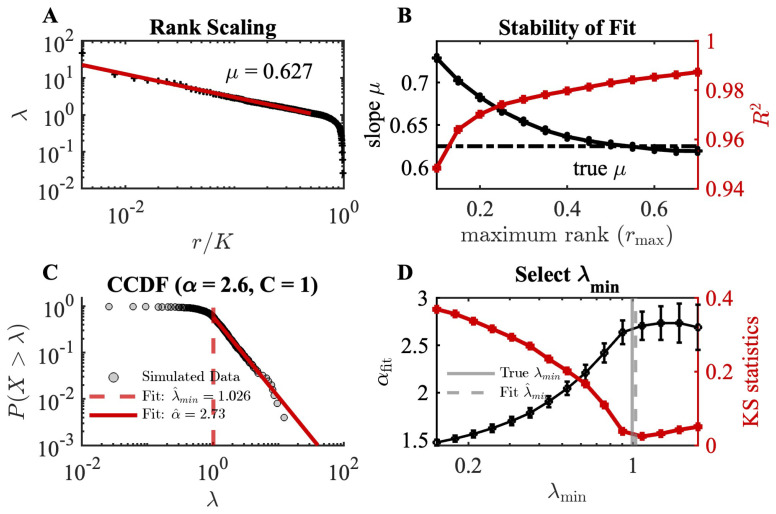
Applying the fitting strategy on a toy model where we know the lower cutoff and scaling exponent α. (**A**) A plot of λ vs. scaled rank r/K, demonstrating approximate power-law scaling λ∼${\left(r/K\right)}^{-\mu }$. Red line indicates a linear fit to the first half of the eigenvalues, which has a slope of μ=0.627. (**B**) The slope (μ, black) and the quality of fit ($R^{2}$, red) obtained from a linear fit using observations from rank 1 to the maximum rank $r_{\max}$. Slope depends on the selection of cutoff, despite high values of $R^{2}$ throughout. Dashed black line indicates true value of μ. (**C**) Complementary cumulative distribution of λ (CCDF), showing best-fit $\lambda_{\min}$ and α. (**D**) Plot of $\alpha_{\mathrm{fit}}$ (black) and the KS statistic of the fitted power law (red) vs. $\lambda_{\min}$. Selection of lower cutoff ($\lambda_{\min}$) is based on minimizing the KS statistic (red) and determines the best-fit scaling exponent α (see Appendix A.1).

In the pRG analysis, the eigenvalue spectrum of each size cluster *K* is sorted by rank and then averaged. To replicate this procedure, we generated K=250 samples of λ from the toy model for each of the $N_{K}=4$ “clusters.” These samples were sorted from largest to smallest, then averaged at each rank to obtain the λ versus rank plot as shown in Figure 2A. We first fit λ∼$r^{-\mu }$ using linear regression of logλ against logr. To avoid including the exponential tail, we fit over a selected range, from rank 1 to the maximum rank $r_{\max}$. Regardless of cutoff, the fit quality appeared high (red line, Figure 2B, $R^{2}>0.94$), but the scaling exponent μ depended on the cutoff value, decreasing from 0.73 to 0.63, with the true value of μ=0.625. Thus, even with a high $R^{2}$ value, the fitted power-law exponent may not be accurate.

Alternatively, we fit the distribution of eigenvalues, ρ(λ)∼$\lambda^{-\alpha }$ (Figure 2C), following the prescription described previously [28] (also see Section 2.2 and Appendix A.1). Here, the minimum cutoff value ($\lambda_{\min}$) is determined by calculating the KS statistic between empirical observation and a true power law with each possible cutoff and selecting the cutoff that minimizes KS (Figure 2D). We find ${\hat{\lambda }}_{\min}=1.026$, obtaining a scaling exponent $\alpha^{\mathrm{fit}}=2.73$, which are close to the true values $\lambda_{\min}=0.99$ and $\alpha^{\mathrm{true}}=2.6$. Translating the fitted value of α to obtain the rank-scaling exponent, we find that $\mu^{\mathrm{fit}}=0.65$, close to the true value and lacking the ambiguity of heuristic selection of the maximum rank cutoff (Figure 2B).

Finally, we used a resampling approach to determine goodness of fit (see Appendix A for details). In this test, the KS statistic between the observations and the fitted model is compared to that obtained by sampling from a true power law and fitting the same model, with a *p*-value calculated as the fraction of resampled KS statistics that exceed the empirically observed value. A *p*-value greater than some threshold (here, 0.1) indicates that a power-law fit cannot be ruled out. For the toy model, we obtained *p*-values ranging from 0.4 to 0.8 at the best-fit parameters across different runs of the simulation, suggesting that a power-law fit is a plausible description of the data.

### 3.2. Estimating μ in a Synthetic Neural Model

Next we test the procedure in a synthetic neural model that exhibits approximate power-law behavior. This controlled setting allows us to systematically vary population size and recording duration while holding all other parameters fixed. Since we now have a “probe” that allows us to reliably reject a power law, we can identify the parameter regimes. Our goal is not to demonstrate that power laws can be confirmed, but rather to identify the minimum data requirements under which scaling behavior—if present—can be detected as statistically consistent with a power law, and conversely, the regimes in which finite sampling leads to cutoff-sensitive fits or outright rejection of the power-law hypothesis.

#### 3.2.1. Population Size Requirements

The synthetic neural model allows us to determine how large of a population must be recorded in order to reliably reveal the scaling of eigenvalues. For instance, in order to quantify eigenvalue scaling in a cluster of size K=128, is it sufficient to record just 256 neurons, or must the population be much larger? To answer this, we performed simulations across a range of total population sizes (N=128,256,512,1024,2048, and 4096), keeping statistical properties of model parameters identical ($J_{ij}$∼$N(0,1/N_{F})$), see Section 2.3.1).

**Figure 3:**
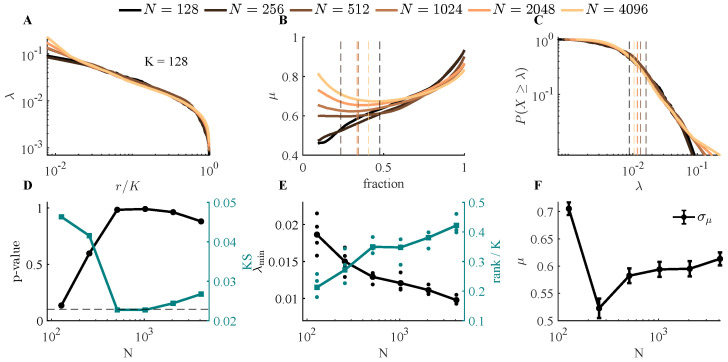
Effect of total population size *N* on the eigenvalue distribution obtained from covariance matrices of clusters of size *K*, using simulated neural population data. Other parameters are fixed (η=3, ϵ=8, $N_{f}=10$, τ=2 s, ΔT=0.02 s, T=600 s). (**A**) For clusters of size K=128, covariance matrix eigenvalues λ are plotted against scaled rank (r/K) for different population sizes (*N*). (**B**) Slope from power-law fit to eigenvalue-vs.-rank plot as a function of the selected cutoff. The dash lines represent the selected $\lambda_{min}$. (**C**) Eigenvalues from (**A**), now plotted as a distribution (CCDF), at each value of *N*. Dashed lines show best-fit $\lambda_{\min}$ for population size (black, N=128 through gold, N=4096). (**D**) Goodness-of-fit, quantified by KS statistic (teal) and corresponding *p*-values (black), as a function of population size. The dashed line indicates p=0.1; p>0.1 indicates that a power-law relationship cannot be ruled out. (**E**) The range of the power-law region expands with increasing population size. For each population size, simulations were repeated four times, and the average cutoff fraction was estimated. The right y-axis represents the fraction of eigenvalues used to fit the power-law distribution. The dots represent results from different runs of simulations. (**F**) The slope of the power-law distribution converges to a value around 0.6 for N>256. Error bars denote $\sigma_{\mu }$, arising from fitting.

For each population size, we performed pRG analysis and then analyzed the eigenvalue distribution for clusters of size K=128. Visually, eigenvalue spectra for size clusters K=128 depend on the size of the total population (Figure 3A), and as with the toy model, we found that power-law fit of eigenvalues versus rank was very sensitive to the choice of cutoff (Figure 3B), especially when the population size is small. Thus, we instead fit the density of eigenvalues (Figure 3C). We found that, at small population sizes, power-law behavior can be rejected for N=128 and 256, while for larger populations, it was not rejected. At larger population sizes, a larger range of eigenvalues appeared to follow power-law behavior, indicated by the smaller minimum eigenvalue cutoff $\lambda_{\min}$ (alternatively, the larger range of rank used for fitting), shown in Figure 3E.

Estimates of μ obtained from fitting also varied with *N* (Figure 3F). Values of μ were stable for populations of size N≥512, but smaller populations yielded different μ values. At all sizes, the uncertainty in μ arising from fitting error was on the order of 0.03. This did not decrease with population size, as might be expected, because the number of eigenvalues used for fitting depends on the cluster size *K*, not the population size *N*.

#### 3.2.2. Cluster Eigenvalues and the Greedy Selection Procedure

In Section 3.2.1, we presented the results of the power-law fitting of the covariance matrix eigenvalues across various population sizes. We observed that a dominant (leading) eigenvalue begins to emerge in the simulated data as the population size increases. To investigate whether this effect could arise from the greedy pairing procedure used in pRG, we examined how clusters are formed when the overall population is very large (e.g., N=8192), while analyzing clusters of fixed size K=128.

**Figure 4:**
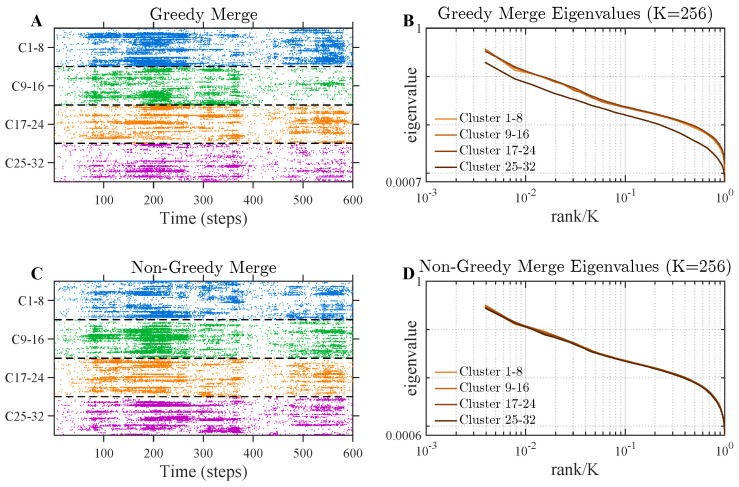
Effect of greedy versus randomized pairing on cluster-level eigenvalue spectra. We simulated a population of N=8192 neurons and applied pRG merging up to cluster size K=256, yielding 32 clusters. Clusters were grouped into four quartiles according to the order in which they were formed during the merging process. For each quartile, we computed the average eigenvalue spectrum of the cluster covariance matrix. (**A**) Activity sorted by cluster under the standard greedy pairing algorithm. Each color indicates a new quartile. (**B**) Eigenvalue spectra of clusters averaged within each quartile. Clusters formed earlier exhibit systematically different eigenvalue spectra than those formed later, with later clusters showing reduced correlation structure. (**C**) Activity sorted by cluster under a modified procedure in which neurons are randomly selected and paired with their most correlated partner. Each color indicates a new quartile. (**D**) Eigenvalue spectra of clusters averaged within each quartile. Ordering-dependent spectra is substantially reduced.

Under the greedy algorithm, neuron pairs are merged in order of highest correlation. As a result, the earliest-formed clusters may concentrate highly correlated neurons, whereas later clusters are formed from progressively weaker correlations. To test this intuition, we applied pRG to the large simulated population and grouped the resulting K=128 clusters according to the order in which they were formed (first quarter, second quarter, third quarter, fourth quarter) (Figure 4A). Each quartile is composed of eight clusters, which may not ultimately be paired together. We found that the eigenvalue spectra differed systematically across these groups, with earlier clusters exhibiting stronger concentration of correlation structure (Figure 4B).

To assess whether this effect arises from greedy ordering, we implemented a modified pairing procedure in which a neuron is selected at random and then merged with its most correlated partner; this process is repeated until no further merges are possible at that scale. This preserves correlation-based pairing while reducing the systematic ordering bias introduced by the greedy strategy. Under this modified procedure, the discrepancy across cluster groups was substantially reduced (Figure 4C,D).

#### 3.2.3. Recording Duration Requirements

**Figure 5:**
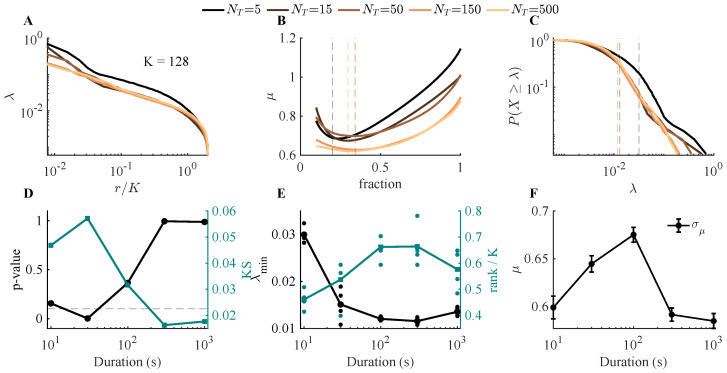
Effect of recording duration on scaling. As in Figure 3, parameters are fixed (N=1024, η=3, ϵ=8, $N_{f}=10$, τ=2 s, ΔT=0.02 s), and only the total simulation length *T* is varied. Because the latent dynamics have characteristic timescale τ, the effective number of independent samples is T/τ. (**A**,**C**) For short recordings (T/τ=5,15,50), eigenvalue spectra deviate from power-law behavior ((**A**), λ vs. rank; (**C**), complementary cumulative distribution of λ). (**B**) Linear fits of eigenvalue vs. rank are sensitive to cutoff choice at small T/τ. (**D**) Statistical tests reject a power law for short durations, while for T/τ≳50, *p*-values approach 1, consistent with a plausible power-law hypothesis. (**E**,**F**) Estimated cutoff $\lambda_{\min}$ (**E**) and scaling exponent μ (**F**) as functions of T/τ, showing convergence once recording duration is sufficiently long.

To investigate how the duration of the recording influences the observed eigenvalue scaling, we fixed the population size to N=1024, which is sufficient to yield reliable estimates of μ based on Figure 3, and then varied the total duration (*T*) of the simulation. The simulated population activity is driven by latent dynamics with a characteristic timescale of τ (2 s), giving an effective number of independent samples of approximately T/τ. For short recordings, the eigenvalue spectra for cluster size K=128 varied, visible both in eigenvalue vs. rank (Figure 5A) and distribution plots (Figure 5C), and fits of eigenvalue against rank are very sensitive to cutoff choices (Figure 5B). Statistical testing (Figure 5D) rules out a power law at these durations.

Eigenvalue spectra for duration of T/τ=150 and T/τ=300 are nearly indistinguishable, and both survive the rejection test (Figure 5D). We show the cutoffs and scaling exponents determined at each value of T/τ in Figure 5E,F, again with inconsistent estimates at short durations that converge once T/τ is long enough. In summary, the smaller the sample size, the poorer the power-law fit. For this system, a minimum duration of T/τ=150 was necessary, and simulation beyond that time did not change conclusions.

We further demonstrate in Appendix B that this duration dependence is not specific to the latent variable model by applying the same procedure to a contact process at criticality.

#### 3.2.4. Time Binning Affects Goodness of Power-Law Fitting

We discovered that bin size is one of the factors that may increase the *p*-value. As bin size increases, each bin accumulates more spikes, effectively acting as a low-pass filter over the neural time series. This temporal averaging suppresses fast independent noise while preserving the slow correlated structure across neurons, causing $\hat{\Sigma }$ to become more and more dominated by the true signal covariance $\Sigma_{\mathrm{signal}}$.

**Figure 6:**
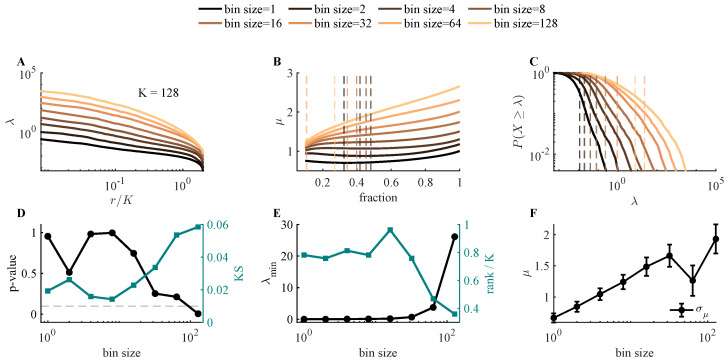
Effect of bin size on power-law fit quality and estimated exponent. (**A**) Eigenvalues as a function of rank for different bin sizes. (**B**) Estimated slope as a function of the lower cutoff, showing sensitivity of the fit to the selected range. (**C**) Complementary cumulative distribution function (CCDF) of eigenvalues. (**D**) KS statistic (teal) and corresponding *p*-value (black) as a function of bin size. (**E**) Fraction of the spectrum identified as power law as a function of bin size. (**F**) Estimated slope μ grows approximately linearly with log(binsize).

In Figure 6, the simulation has 1024 neurons, and the time constant for the latent variable is 100 times the time step. The true signal is the shared fluctuation projected onto neurons through the weight matrix *J* by the latent field *h*. When the bin size increases, the covariance structure starts to be dominated by the underlying correlation structure of *h* when timestep≪binsize<τ. However, when the bin size increases, two mechanisms start to influence the quality of the power-law fit to the eigenvalue spectrum of Σ. First, the number of available samples decreases. Second, when ΔT>τ, the temporal averaging begins to eliminate the slow-varying structure. These two mechanisms set an upper bound on usable bin sizes. However, within the feasible range, μ increases monotonically with bin size (Figure 6F). In real neural data, bin size must be chosen before analysis, and this choice will influence the estimated exponent. Rather than identifying an optimal bin size, our surrogate modeling framework characterizes how μ is expected to vary with binning, enabling more principled interpretation of exponents estimated from real data.

Taken together, these simulations identify the conditions under which power-law fits are reliable. When the population size *N* or the number of independent samples (T/τ) is insufficient, the power-law hypothesis may be rejected even when the generative model exhibits scaling. As both quantities increase, eigenvalue spectra become statistically consistent with a power law, though this should be read as failure to reject rather than confirmation of a true power-law mechanism.

### 3.3. Application to Experimental Neural Recordings

Having established data-dependent constraints in simulation, we next apply the same fitting framework to experimental recordings from [29,30]. Our goal is to assess which datasets fall into regimes where power-law behavior can be statistically ruled out, and which fall into regimes where the data are statistically consistent with a power law and exponent estimates are meaningful.

**Figure 7:**
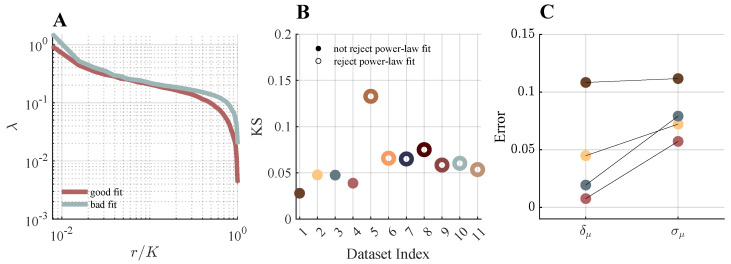
Application of the proposed method to large-scale neural recordings. We examine the quality of power-law scaling and fitting errors in experimental datasets from [29,30]. (**A**) Eigenvalue spectra at K=128 are shown for brain areas from the [29] datasets (red: FrMoCtx; blue: TH), illustrating examples of spectra with good and poor power-law fits. (**B**) KS statistics for each analyzed dataset. Filled circles denote cases with *p*-value ≥0.1, where the power-law hypothesis is not rejected, whereas a *p*-value <0.1 indicates the rejection of power-law hypothesis. (**C**) Subsampling error ($\delta_{\mu }$) and asymptotic error ($\sigma_{\mu }$) are reported for datasets in which the power-law hypothesis is not rejected.

In Figure 7A, we show representative eigenvalue spectra (for K=128) from two brain areas recorded in the same animal with comparable neuron counts, illustrating cases with visibly different scaling behavior.

Figure 7B summarizes the KS statistics and corresponding *p*-values obtained for each dataset. Rather than interpreting these results as a strict binary classification, we view the *p*-value as a measure of compatibility with a power-law model under finite sampling. Several datasets yield *p*-values below the nominal threshold (0.1), indicating that deviations from a fitted power law are larger than expected under the model. Others yield *p*-values above this threshold, placing them in a regime where the data are statistically consistent with a power law. Importantly, the distinction between these regimes is often quantitative rather than qualitative, with many datasets lying close to the rejection boundary. We emphasize that failure to reject does not constitute confirmation of a power law; rather, it indicates that the available data do not provide sufficient evidence to rule it out.

In Figure 7C, we compare the asymptotic uncertainty in μ (arising from the maximum-likelihood fit) with a subsampling-based estimate of variability obtained by dividing each dataset into four non-overlapping temporal segments and refitting independently. In datasets that are statistically consistent with a power law, the asymptotic fitting error typically dominates over subsampling variability. This suggests that, in these recordings, uncertainty is primarily governed by the limited number of eigenvalues contributing to the fitted tail rather than by temporal heterogeneity.

As shown in previous sections, simulations based on latent variable models reveal that neuronal population size (Figure 3) and recording duration (Figure 5) strongly influence power-law fitting. To further evaluate these effects in experimental neural recording datasets, we selected the neurons from the frontal motor cortex (FrMoCtx) in Table 2 as a test case, as it was not rejected under the power-law hypothesis in our previous analysis (Figure 7).

**Figure 8:**
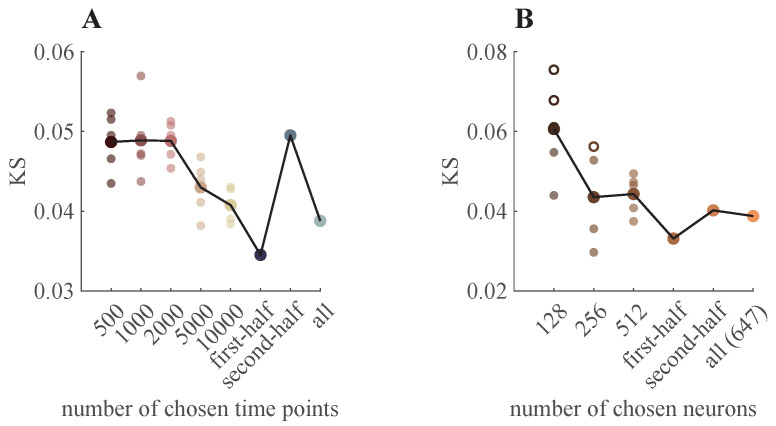
Time and population size effect on power-law goodness-of-fit in dataset #4 (Table 2). (**A**) KS statistic is plotted as a function of the number of sampled time points. For the first five conditions (500–10,000 time points), five independent random subsamples were drawn; small dots represent individual repetitions, and large dots denote their mean. The remaining conditions (first half, second half, and all data) correspond to fixed partitions of the recording. Filled circles indicate non-rejection of the power-law hypothesis (p≥0.1), whereas hollow circles indicate rejection (p<0.1). (**B**) KS statistic is plotted as a function of the number of neurons included in the analysis. For the first three condition (128, 256, and 512 neurons), five independent random subsamples were drawn. The plotting scheme and marker convention are identical to those in (**A**).

In Figure 8A, we compare the KS values across different time-point sampling conditions. For each condition, we randomly selected 500 time points and computed the corresponding KS and *p*-value for the power-law fitting, repeating this procedure five times. The same procedure was applied for 1000, 2000, 5000 and 10,000 time points. In addition, we divided the dataset into two halves and evaluated the KS and *p*-values separately for the first and second halves. The results show that different numbers of chosen time points within the same dataset can lead to markedly different fitting qualities.

Figure 8B shows that varying the number of neurons also affects fitting quality. Specifically, we followed the same procedure used to examine the time-point effect, but instead varied the number of randomly selected neurons.

## 4. Discussion

In this work, we first reformulated eigenvalue–rank fitting as a distributional fitting problem. Rather than relying on linear trends in log–log space, we fit the distribution of eigenvalues and used a Kolmogorov–Smirnov criterion to determine the lower cutoff ${\hat{\lambda }}_{\min}$ in a principled manner. This approach avoids arbitrary cutoff selection and provides a way to define the region of the spectrum most consistent with a power-law model.

We then examined the behavior of this fitting procedure in simulations, where both population size and recording duration could be systematically varied. In a generative model known to produce approximate scaling in the large-data limit, we found that reliable estimation of the scaling exponent μ in a cluster of size *K* required a minimum population size that was about 4K. Above this threshold, exponent estimates stabilized and were consistent across repeated simulations. Below it, the fitted exponent varied substantially, and power-law behavior could often be rejected. Moreover, we found that at fixed cluster size *K*, while the portion of the spectrum following a power law expands somewhat as the total population *N* increases, the number of eigenvalues contributing to the fit is at most *K*, and so does not increase substantially. As a result, the asymptotic error from fitting with finite samples of data remains large.

We further found that recording duration must be sufficiently long to reliably estimate μ. In our model, the latent dynamics evolved on a characteristic timescale τ, so the relevant sampling parameter was the effective number of independent samples, T/τ. When T/τ was comparable to or smaller than the cluster size *K* used for eigenvalue estimation, fits were sensitive to cutoff selection. Once T/τ substantially exceeded *K*, the eigenvalue spectrum of a given cluster changed little with further increases in recording duration.

Next, we examined the effect of time binning. We and others have previously shown that μ is sensitive to the bin size [3,36]. Here we found that varying the bin size in the simulated data substantially altered the apparent scaling region as well. Thus, time discretization must be carefully considered when comparing datasets, as it can shift recordings between regimes of apparent scaling and rejection.

Finally, we applied the same fitting framework to 11 experimental neural datasets. Under our predefined criterion (p<0.1), four datasets were statistically compatible with a power-law model, six were narrowly rejected but exhibited near-scaling behavior, and one clearly deviated from power-law structure. Subsampling analyses revealed that the KS statistic and rejection outcomes depended systematically on both population size and recording duration within the same dataset. These dependencies closely paralleled the simulation results, which also showed smaller KS values as the population size or recording duration increased. Taken together, these results demonstrate that eigenvalue scaling estimates are strongly conditioned on population size, effective recording duration, and preprocessing choices, and that simulation-based calibration provides a useful framework for interpreting empirical findings.

Our statistical framework tests whether deviations from a fitted power law are large enough to reject the power-law hypothesis (p<0.1). If p≥0.1, this only indicates statistical compatibility under finite sampling. In our simulations, the generative model exhibits power-law scaling in the large-data limit. Increasing *N* and *T* therefore moves the system from a regime where scaling can be rejected to one where it becomes statistically consistent with the data. We emphasize that our results only establish minimum data requirements for detecting scaling rather than constituting a definitive test. Furthermore, our analysis does not perform explicit model comparison against common alternatives (e.g., log-normal), which are difficult to distinguish from power laws under finite-sample conditions. Therefore, throughout, we interpret power-law plausibility cautiously, emphasizing the data regimes in which power-law behavior can be ruled out and the regimes in which the available data are insufficiently informative to distinguish candidate heavy-tailed models.

### Implications for Neural Data Analysis

**Estimation of error:** Often, uncertainty in estimates of μ has been obtained by independently fitting a subset of the neural data, usually by dividing into quarters or fifths. We directly compared this error estimate ($\delta_{\mu }$) to the uncertainty derived by fitting a finite data sample ($\sigma_{\mu }$) in experimental data and found that $\delta_{\mu }$ was smaller than $\sigma_{\mu }$ in three of four experimental recordings. This is supported by our analysis of the dependence of μ on recording duration *T*, which showed high stability above some minimum duration *T*. These results suggest that the dominant source of error arises from fitting, not from non-stationarity in the neural recordings, and in future studies, both should be quantified.
**Alternative to greedy merge:** While sampling larger populations increases the power of the pRG analysis, our analysis suggests that the greedy-merge strategy introduces a systematic ordering bias into the observed eigenvalue spectra. Under the greedy algorithm, the earliest-formed clusters concentrate the most highly correlated neurons, while later clusters are formed from progressively weaker correlations. This leads to systematic differences in eigenvalue spectra across clusters that reflect merge order rather than underlying structure. We implemented a modified procedure in which the seed neuron is selected randomly and then merged with its most correlated partner, repeating until no further merges are possible. This preserves correlation-based pairing while substantially reducing the ordering-dependent variation across cluster groups, and we suggest it as a more principled alternative to the standard greedy strategy.
**pRG and fMRI:** To date, most pRG analyses have been performed on large-scale electrophysiology and calcium imaging datasets, where hundreds to thousands of neurons are recorded simultaneously. One data modality in which pRG analysis is underexplored is functional magnetic resonance imaging (fMRI). Standard processing leads to the brain being divided into approximately 91,000 spatial components [37], termed “grayordinates”, providing an extremely high-dimensional readout of brain activity that is challenging to analyze. While these data feature a lower signal-to-noise-ratio than most other neuroimaging modalities and operate on a relatively slow timescale (one image per 2 s), fMRI can noninvasively monitor human brain activity, and the derived metrics of networks may allow researchers to measure quantitatively differences in brain dynamics in individuals.

A key limitation has been obtaining sufficient temporal samples to carry out pRG analysis, which by our results here, suggests a requirement of T/τ∼*K* for the scale *K* of analysis. Recent advances in fMRI techniques have led to an approach known as precision functional mapping (PFM) [38]. This approach highly samples individual participants so that analyses can be performed on the level of individual participants as opposed to on group-level data. PFM studies often will scan each participant across multiple sessions such that there are approximately 90 min of low-motion data. This often leads to around 2300 frames within the time series for each participant. Autocorrelation in fMRI decays on a timescale of around five frames (or approximately 8.75 s) [39]. As such, PFM fMRI datasets tend to have a T/τ value of 460, plausibly enabling analysis of clusters up to about K=256.

There remain several technical challenges for pRG of fMRI datasets. The high spatial dimensionality increases the influence of the order in which units are selected; the revised coarse-graining suggested here may be crucial in fMRI pRG analysis. Because fMRI lacks a “silence” signal, some metrics of criticality in pRG may not be available, most notably free energy. As such, the two most valuable metrics for interpreting critical dynamics in pRG of fMRI data are covariance eigenvalue spectrum decay and variance scaling.

## 5. Conclusions

If the critical brain hypothesis is to be taken seriously as a quantitative framework, then the scaling exponents that characterize neural activity must be measured with rigor. Apparent power-law behavior cannot rest on visual linearity or isolated fits; it requires clearly defined metrics, reproducible cutoff procedures, and explicit reporting of sampling and preprocessing constraints to enable meaningful comparisons across datasets and modalities. The calibration framework developed here provides one step toward that goal by mapping exponent stability onto population size, recording duration, and analysis choices. More broadly, generative surrogate models like the dynamical latent variable model can benchmark the reliability and limits of future scaling analyses.

## Figures and Tables

**Table 1:** Summary of symbols and parameters used in pRG covariance analysis.

Symbol	Values	Meaning
*K*	64,128,256,…	Cluster size
*N*	256,512,1024,…	Population size
$N_{K}$	1,2,4,…	Number of clusters
λ	Range	Eigenvalues
*r*	1,…,K	Rank of eigenvalue
μ	0.5–1.5	Scaling parameter, λ∼$r^{-\mu }$
α	$1+\frac{1}{\mu }$	Scaling parameter, ρ(λ)∼$\lambda^{-\alpha }$
$\lambda_{\min}$	Range	Eigenvalue cutoff (minimum value/maximum rank)
*T*	Minutes to hours	Duration of recording
τ	Seconds	Timescale of activity (model)

**Table 2:** Dataset index and corresponding data information.

Dataset Index	Brain Area	Number of Neurons	μ	Source
1	CP	249	0.679	[30]
2	CP	534	0.620	[30]
3	MOp	277	0.914	[30]
4	FrMoCtx	647	0.382	[29]
5	CP	390	0.565	[30]
6	CP	454	0.440	[30]
7	MOp	250	0.427	[30]
8	MOp	722	0.500	[30]
9	V1	334	0.323	[29]
10	TH	638	0.343	[29]
11	TH	1878	0.376	[29]

**Figure A1:**
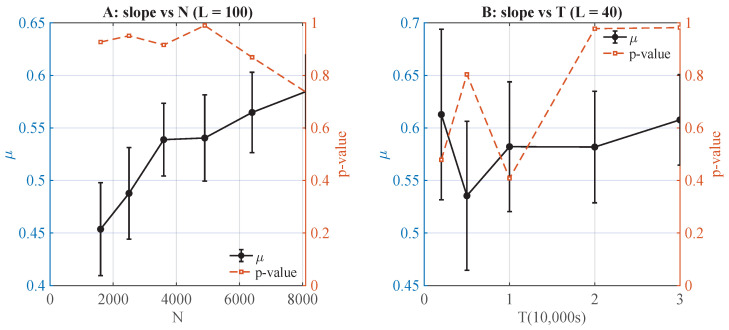
Eigenvalue scaling exponents for a contact process on a 2D lattice of size $N=L^{2}$ with spreading rate 1.6488. (**A**) Estimated exponent μ as a function of randomly subsampled sites *N*. The exponent varies across subsampling sizes but remains consistent with a power law in all cases. (**B**) Estimated exponent μ as a function of recording duration *T* at fixed $N=40^{2}$. Above ≈$10^{4}$ steps, estimates stabilize at a value consistent with Nicoletti et al. [31].

## Data Availability

All data included were previously published and publicly available.

## Appendix A. Detailed Mathematical Procedures

### Appendix A.1. Maximum Likelihood Estimation

We adopt the statistical framework established in [28]. Instead of fitting the eigenvalue-vs.-rank relationship, we fit the probability density function (PDF) of the eigenvalues directly. We assume that for eigenvalues λ greater than some lower bound $\lambda_{\min}$, the probability density follows:

(A1) $$p\left(\lambda \right)=C\lambda^{-\alpha }\;\mathrm{for}\;\lambda \geq \lambda_{\min}$$

where α is the scaling parameter of the distribution, and *C* is a normalization constant. Given the constraint $\int_{\lambda_{\min}}^{\infty }p\left(\lambda \right)d\lambda =1$, the normalized PDF is:

(A2) $$p\left(\lambda \right)=\frac{\alpha -1}{\lambda_{\min}}{\left(\frac{\lambda }{\lambda_{\min}}\right)}^{-\alpha }$$

Given a set of *n* observed eigenvalues $\Lambda =\{\lambda_{1},\ldots ,\lambda_{n}\}$ such that $\lambda_{i}\geq \lambda_{\min}$, the log-likelihood of the data is given by:

(A3) $$L=\sum_{i=1}^{n}\ln p\left(\lambda_{i}\right)=n\ln\left(\alpha -1\right)-n\ln\lambda_{\min}-\alpha \sum_{i=1}^{n}\ln\left(\frac{\lambda_{i}}{\lambda_{\min}}\right)$$

Maximizing L with respect to α yields the Hill estimator, the closed-form Maximum Likelihood Estimator (MLE) [34] for the exponent:

(A4) $$\hat{\alpha }=1+n{\left[\sum_{i=1}^{n}\ln\frac{\lambda_{i}}{\lambda_{\min}}\right]}^{-1}$$

### Appendix A.2. Quantifying Goodness-of-Fit

We follow the goodness-of-fit procedure outlined in [28]. Briefly, we computed a *p*-value based on how often the KS statistic between observed eigenvalues and the fitted model exceeded the KS statistic between finite observations from a true power law with a model fitted to those observations; when the *p*-value is *smaller* than some threshold, a power law can be rejected. We emphasize that this does not compare the power law to alternative models, but does establish that a power law could not be ruled out.

More precisely, we determine $\lambda_{\min}$ algorithmically by minimizing the distance between the empirical data and the theoretical power-law model. Let S(λ) be the empirical cumulative distribution function (CDF) of the observed data for observations $\lambda \geq \lambda_{\min}$, and let P(λ) be the CDF of the theoretical power-law model with the best-fit $\hat{\alpha }$ for that specific $\lambda_{\min}$. We quantify the distance using the Kolmogorov–Smirnov (KS) statistic *D*:

(A5) $$D=\underset{\lambda \geq \lambda_{\min}}{\sup}\left|S\left(\lambda \right)-P\left(\lambda \right)\right|$$

Following [28], our procedure is as follows:

1. We iterate through each unique eigenvalue in the dataset to serve as a candidate $\lambda_{\min}$.
2. For each candidate $\lambda_{\min}$, we estimate $\hat{\alpha }$ via MLE and calculate the KS statistic $\hat{D}$.
3. We select ${\hat{\lambda }}_{\min}$ that minimizes $\hat{D}$.
4. Surrogate data is generated from a model matching the number of samples in the dataset and fixing $\lambda_{\min}={\hat{\lambda }}_{\min}$ and $\alpha =\hat{\alpha }$. On each iteration *i* ($i=1\ldots N_{S}$), the surrogate dataset is refit, and the KS statistic between the surrogate data and the fitted surrogate data model is calculated ($D_{i}$).
5. We calculate a *p*-value as the fraction of times that the empirical KS statistic $\hat{D}$ exceeds that of the surrogate models: $\sum_{i}\left(\hat{D}>D_{i}\right)/N_{S}$.

The *p*-value obtained indicates whether a power law can be *rejected*, not whether it is confirmed. While the choice of threshold is arbitrary, we choose a conservative value of 0.1 for our threshold for rejecting power-law behavior. Note that, in this framework, lower thresholds are more permissive.

### Appendix A.3. Transformation to Rank Scaling and Error Propagation

The MLE procedure produces the distribution exponent α. To compare our results with standard pRG literature, we must transform the complementary cumulative distribution function (CCDF) scaling exponent α back to the rank-scaling exponent μ.

The rank *r* of an eigenvalue λ is proportional to the CCDF, P(X≥λ). Integrating the PDF:

(A6) $$r\left(\lambda \right)\propto \int_{\lambda }^{\infty }\lambda^{-\alpha }d\lambda \propto \lambda^{-(\alpha -1)}$$

Inverting this relationship to express λ as a function of rank yields:

(A7) $$\lambda \propto r^{-\frac{1}{\alpha -1}}$$

Comparing this to the definition $\lambda \propto r^{-\mu }$, we derive the transformation:

(A8) $$\mu =\frac{1}{\alpha -1}$$

Finally, we quantify the uncertainty in our estimate. The standard error of the MLE estimator $\hat{\alpha }$ is asymptotically normal with standard deviation:

(A9) $$\sigma_{\alpha }=\frac{\hat{\alpha }-1}{\sqrt{n}}$$

where *n* is the number of samples in the tail ($\lambda \geq \lambda_{\min}$). Using the Delta method, we approximate the error for μ:

(A10) $$\sigma_{\mu }\approx \left|\frac{d\mu }{d\alpha }\right|\sigma_{\alpha }=\left|\frac{-1}{{\left(\alpha -1\right)}^{2}}\right|\frac{\alpha -1}{\sqrt{n}}$$

This simplifies to a concise expression for the standard error of the rank scaling exponent:

(A11) $$\sigma_{\mu }=\frac{\mu }{\sqrt{n}}$$

This framework provides a parameter-free and statistically rigorous estimate of μ and its associated uncertainty, robust to the selection biases inherent in linear regression methods.

## Appendix B. Eigenvalue Scaling in a Critical Model

The results in the main text are derived from simulations of a dynamic latent variable model, which is not a critical system. To demonstrate that our estimation procedure generalizes beyond this setting, we applied it to a qualitatively different model: a contact process on a square 2D lattice tuned to criticality. Following Nicoletti et al. [31], we used periodic boundary conditions and set the spreading rate to the numerically estimated critical value $\lambda_{c}\approx 1.6488$ [31,40].

In all cases, power-law behavior could not be ruled out, though the estimated exponent varied with both the number of sampled sites and recording duration (Figure A1). The dependence of the estimated exponent on system size (Figure A1A) reflects the fact that subsampling a different number of sites from the same lattice yields different covariance structures; we do not attempt to interpret this dependence further here. At fixed $N=40^{2}$, exponent estimates converged as *T* increased, stabilizing at a value consistent with Nicoletti et al. [31] (Figure A1B). This mirrors the duration dependence observed in the latent variable model, suggesting that the qualitative procedure for determining sufficient recording duration is not specific to a particular model class.
